# Supplementary material for: The cryptic seismic potential of the Pichilemu blind fault in Chile revealed by off-fault geomorphology
Source: Nat Commun. 2022 Jun 11;13:3371. doi: 10.1038/s41467-022-30754-1 (PMC9188598; doi:10.1038/s41467-022-30754-1)
Supplement: Supplementary file 1 — Supplementary Information [file 41467_2022_30754_MOESM1_ESM.pdf]

Supplementary Information: The cryptic seismic potential of the Pichilemu blind fault in Chile revealed by off-fault geomorphology

J. Jara-Muñoz[1], D. Melnick[2], S. Li[3], A. Socquet[4], J. Cortes-Aranda[5], D. Brill[6], M.R. Strecker[1]

[1] University of Potsdam, Department of Earth Sciences, Karl-Liebknecht-Str. 24-25, Potsdam, Germany
[2] Austral University of Chile, Institute of Earth Sciences, Edificio Pugín, Campus Isla Teja, Valdivia, Chile
[3] Chinese Academy of Sciences, State Key Laboratory of Lithospheric Evolution, Institute of Geology and Geophysics, No. 19, Beitucheng Western Road, Beijing, China.
[4] University of Grenoble Alpes, University Savoie Mont Blanc, CNRS, IRD, University of Gustave Eiffel, 1381 Rue de la Piscine, Grenoble, France.
[5] Universidad de Concepción, Department of Earth Sciences, Víctor Lamas 1290, Concepcion, Chile.
[6] University of Cologne, Institute of Geography, Otto-Fischer-Straße 4, Cologne, Germany.

J. Jara-Muñoz (jara@geo.uni-potsdam.de), D. Melnick (daniel.melnick@uach.cl), S. Li (shaoyangli@mail.iggcas.ac.cn), A. Socquet (anne.socquet@univ-grenoble-alpes.fr), J. Cortes-Aranda (joacortes@udec.cl), D. Brill (brilld@uni-koeln.de), M. R. Strecker (Manfred.Strecker@geo.uni-potsdam.de)

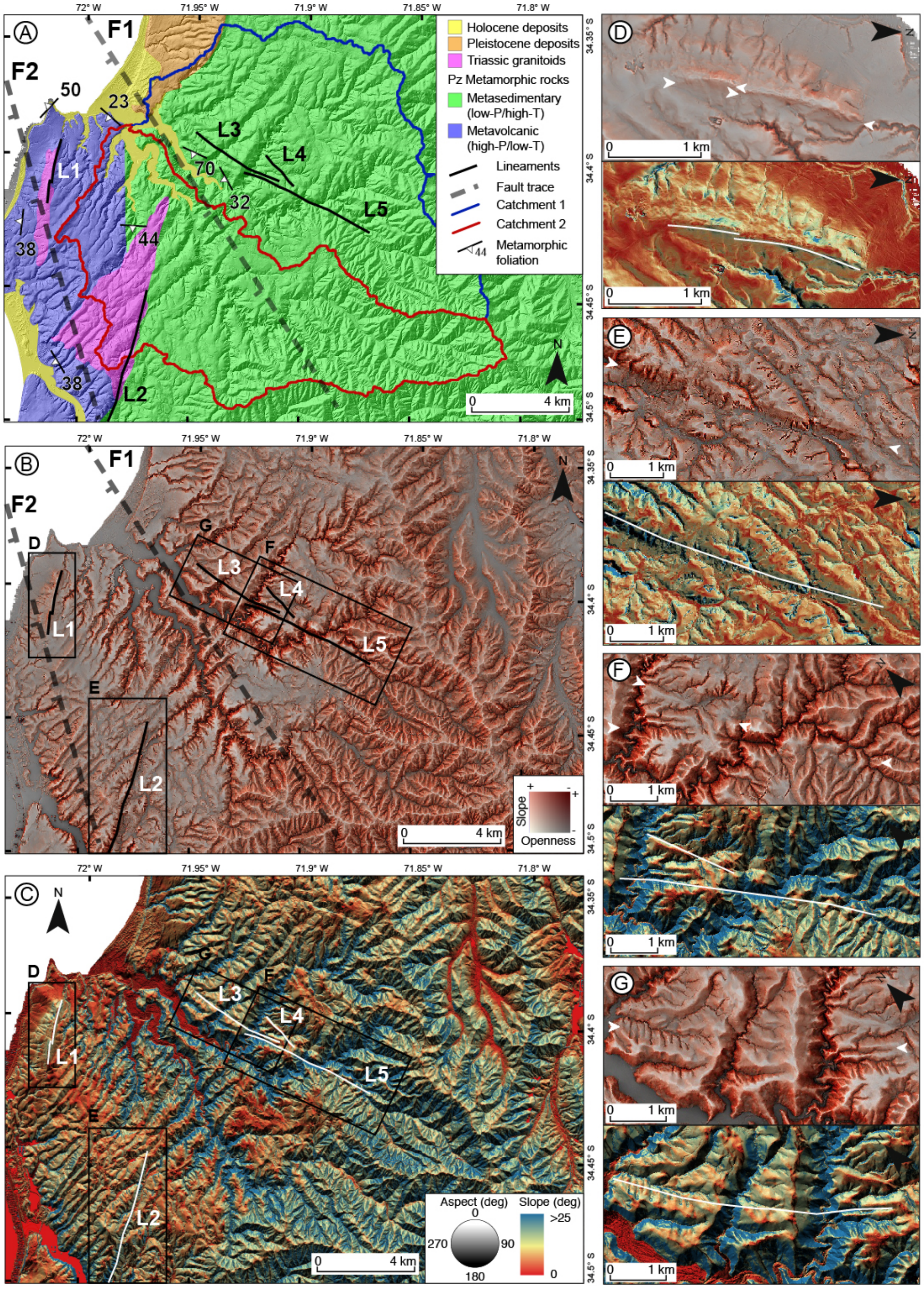

Figure S1: Analysis of lineaments and geological structures using LiDAR Digital Terrain Models. A) Geological map of the PIF area based on[1]. The dashed thick black lines are the F1 and F2 faults, inferred from aftershock seismicity; lineaments are denoted by labels L1 to L5 and represent potential faults that are suspected to reach the surface. Numbers with the dip symbol indicate the dip of metamorphic foliation in degrees. Palaeozoic metamorphic rocks constitute low-pressure/high-temperature (low-P/high-T)

metasedimentary rocks and high-pressure/low-temperature (high-P/low-T) metavolcanic rocks[1]. B) Red-relief map used to map the trace of lineaments. C) Directional slope map. D, E, F and G are close-up views of B and C at the locations of lineaments and are indicated by white arrows (See Section 3.1 for details).

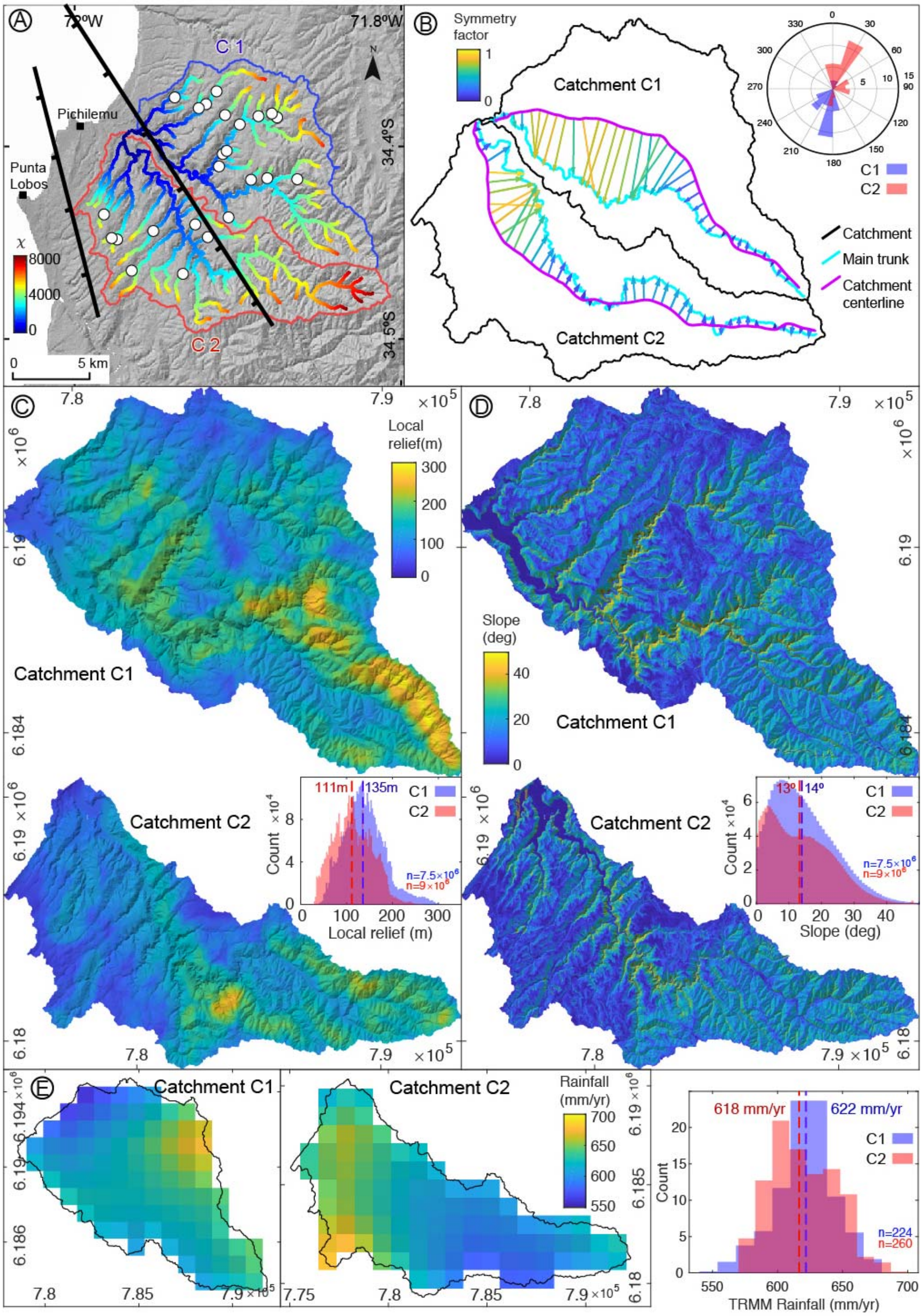

Figure S2: Fluvial catchment analysis. A) Chi-plot of catchments 1 and 2. Chi values were extracted at the location of knickpoints (white dots) to explore the potential relationship between lineaments and knickpoint metrics in Fig. 2B. B) Catchment asymmetry; the

arrows represent vectors color-coded by symmetry factor indicating the distance between the catchment centreline and the main trunk stream (see Methods, Section 4.2 for details). The rose diagram (inset) displays the orientation of the symmetry vectors of catchments C1 and C2. C) Local relief of catchments C1 and C2 calculated using a moving window of a 500 m diameter. The histogram in the inset displays the distribution of local relief of C1 and C2, the y axis displays the number of pixels. The dashed lines are median values. D) Slope maps of catchments C1 and C2. E) TRMM rainfall within each catchment based on a 12-year time series[2]; n corresponds to number of elements.

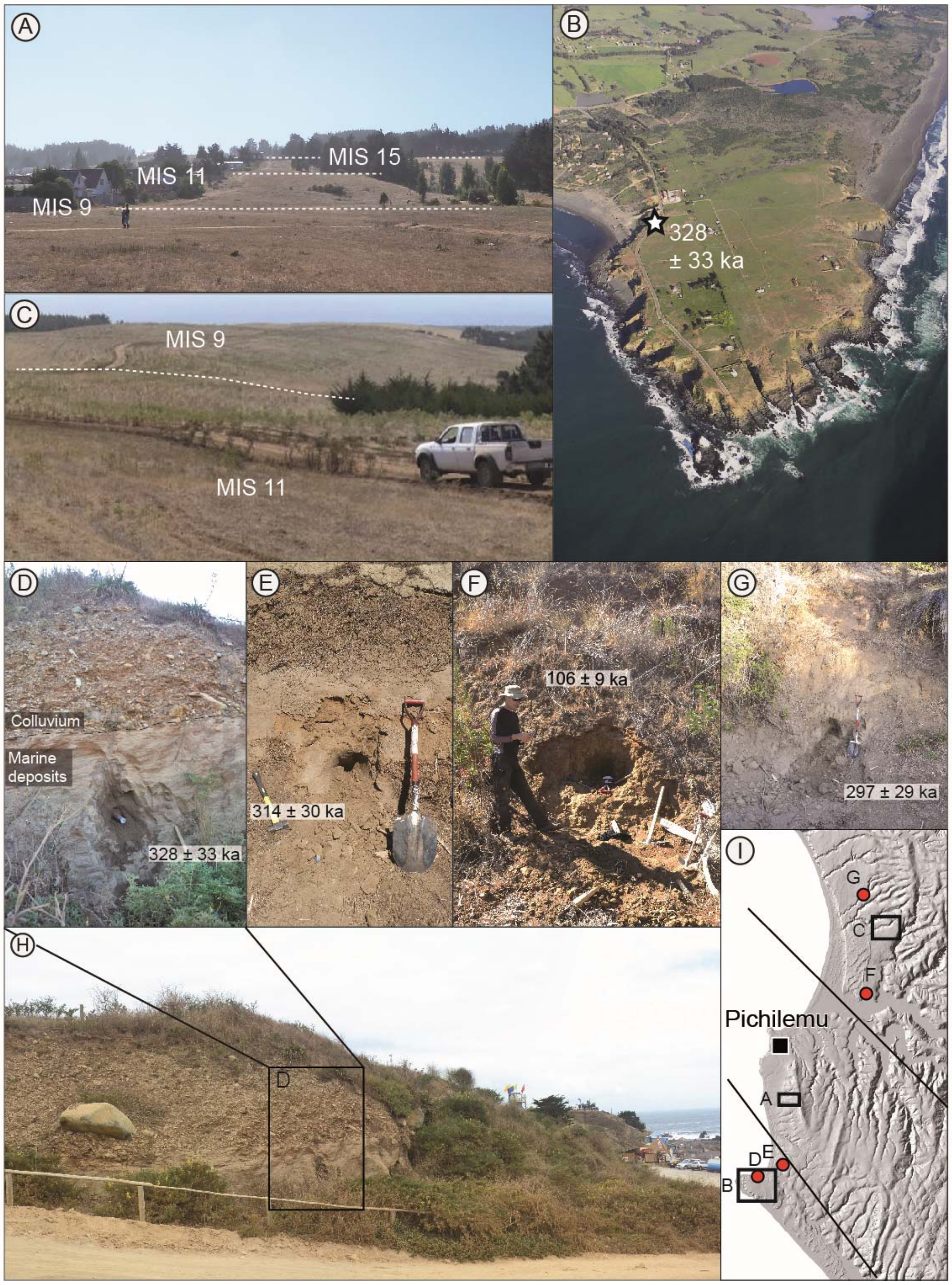

Figure S3: Field views of marine terraces in the PIF area and IRSL sampling sites. A) View of wave-cut marine terraces along the PIF hanging-wall block. **B)** Aerial view of wave-cut terraces at Punta Lobos. **C)** View of wave-built terraces in the PIF footwall block. **D-G)** Outcrop views of sampling sites for IRSL age dating. **H)** Panoramic view of sampling site LOBO-1 (location in B and I). **I)** Index map showing location of field and sampling site views.

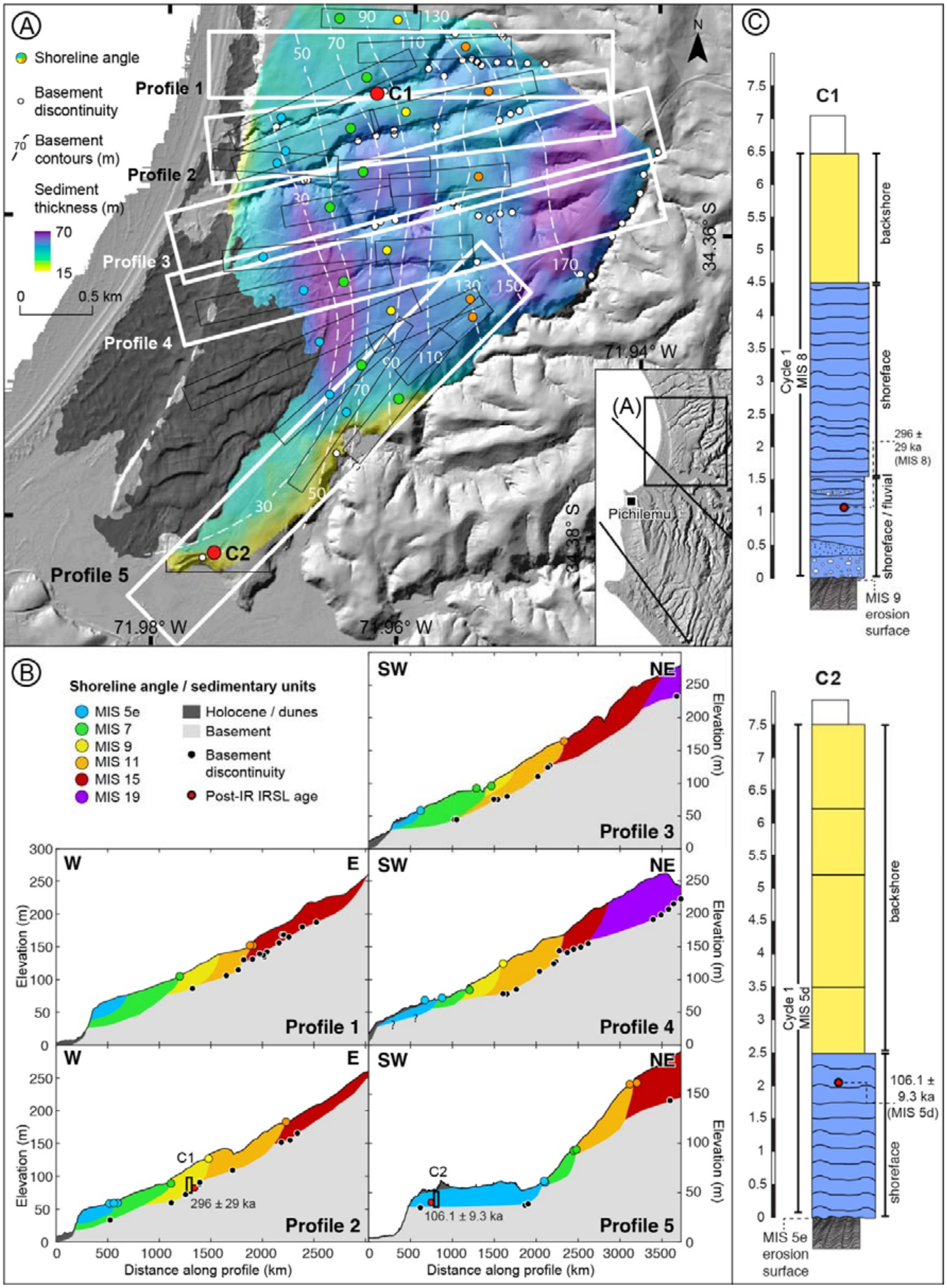

Figure S4: Stratigraphy of wave-built terraces in the footwall block of the PIF. A) Shaded-relief topography from 1-m LiDAR DTM (inset shows the location and main PIF strands). White circles represent field measurements of the basement discontinuity. White

contours display the depth to crystalline basement. White boxes show location of profiles in B. Black boxes are swath profiles used to map shoreline angles marked as circles that are color-coded according to age (See legend in B). C1 and C2 denote locations of stratigraphic sections in C. The dark grey area corresponds to active and fixed dunes. **B)** Geometry of the sedimentary sequences; black dots are field-measurement sites of the basement discontinuity and thickness of the sedimentary cover enclosed by each white box in A. Red dots denote the location of post-IR IRSL ages in B and C. Blue dots are shoreline angles of marine terraces displayed in A and enclosed by each white box. Black rectangles show position of stratigraphic sections in C. **C)** Stratigraphic sections of the wave-built sequence, both comprising a regressive cycle from shoreface to backshore environment, deposited above the bedrock unconformity. Notice that marine terrace levels corresponding to MIS 9 and 5e are formed by sedimentary sequences above an erosion surface and were deposited during subsequent sea-level drop after both highstands (See section 3.2); therefore, we assigned and age of MIS 9 and MIS 5e to these terrace levels and calculated uplift rates.

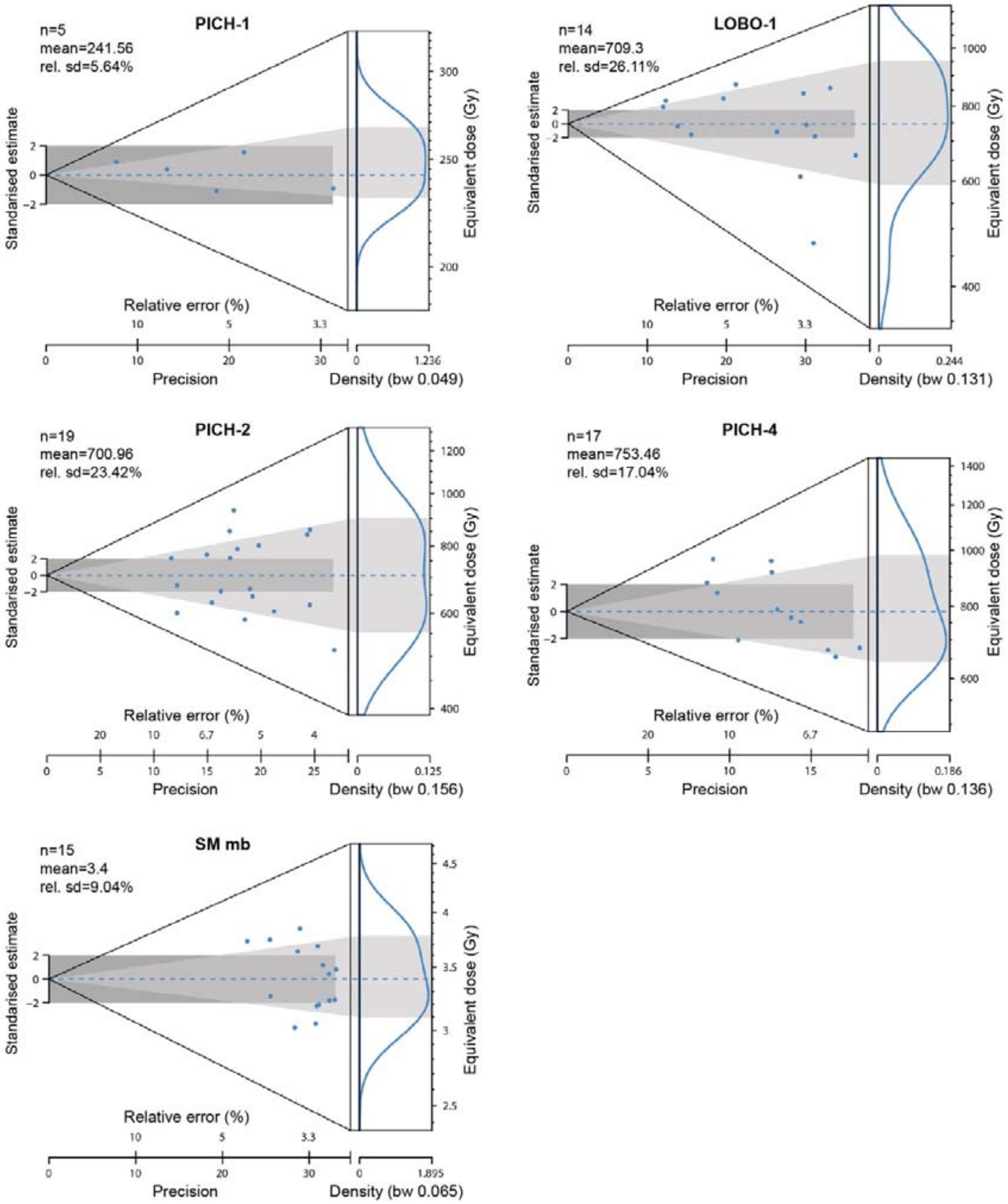

Figure S5: Radial plots of post-IR IRSL equivalent doses. Abanico-type plots of each sample combining radial plots and kernel-density estimates (blue lines). Note that density distributions are unimodal.

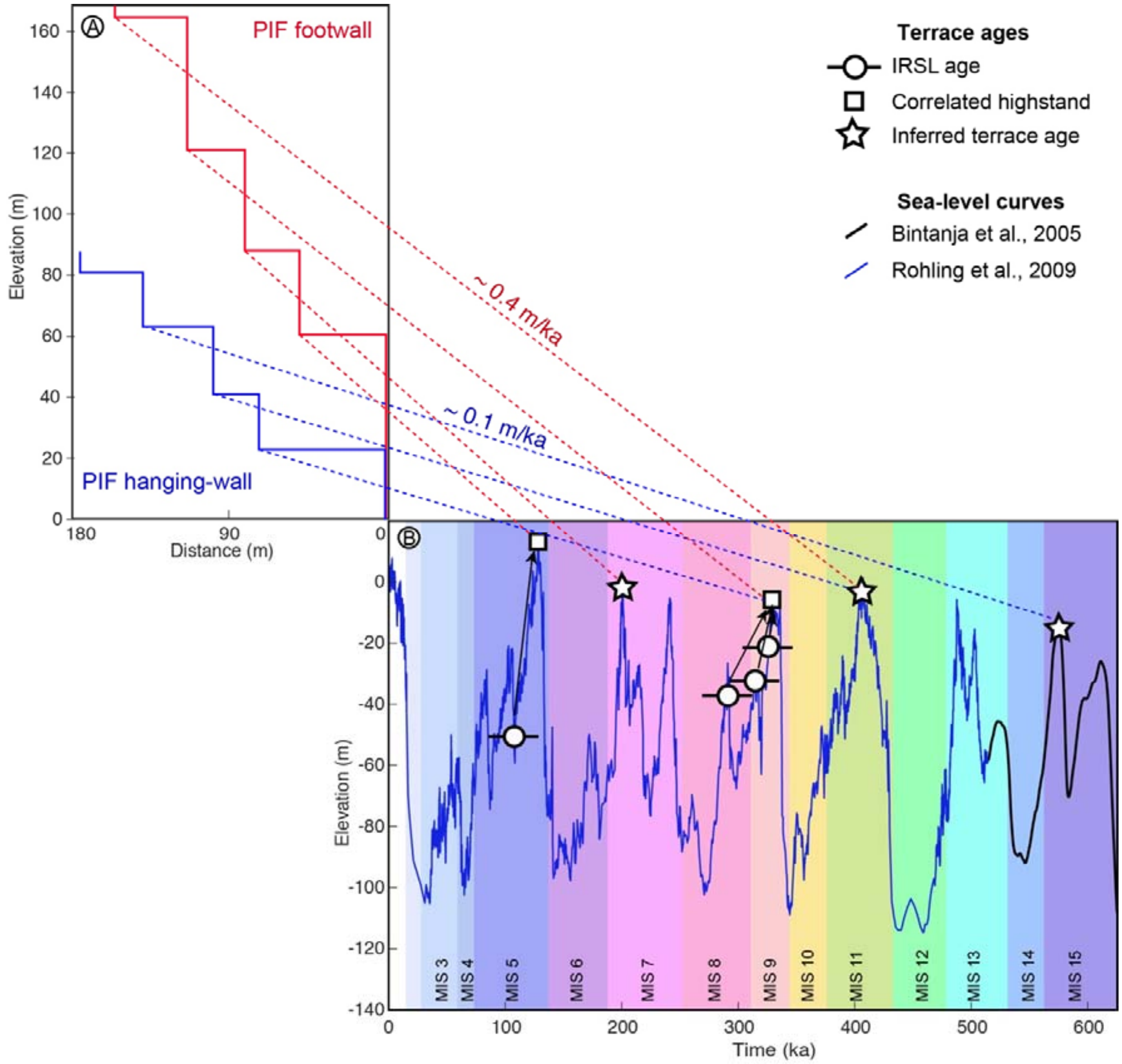

Figure S6: Correlation between marine terraces and Quaternary sea-level highstands. A) Terraces at the northern footwall and southern hanging-wall blocks of the PIF. **B)** Composite sea-level curve based on Rohling et al. [3], between 0 and 500 ka, and Bintanja et al. [4] after 500 ka. Note that uplift rates (slope of dashed lines) are approximately constant for the different levels within each fault block. Error bars indicate the temporal error of IRSL ages (see Table 1 for details).

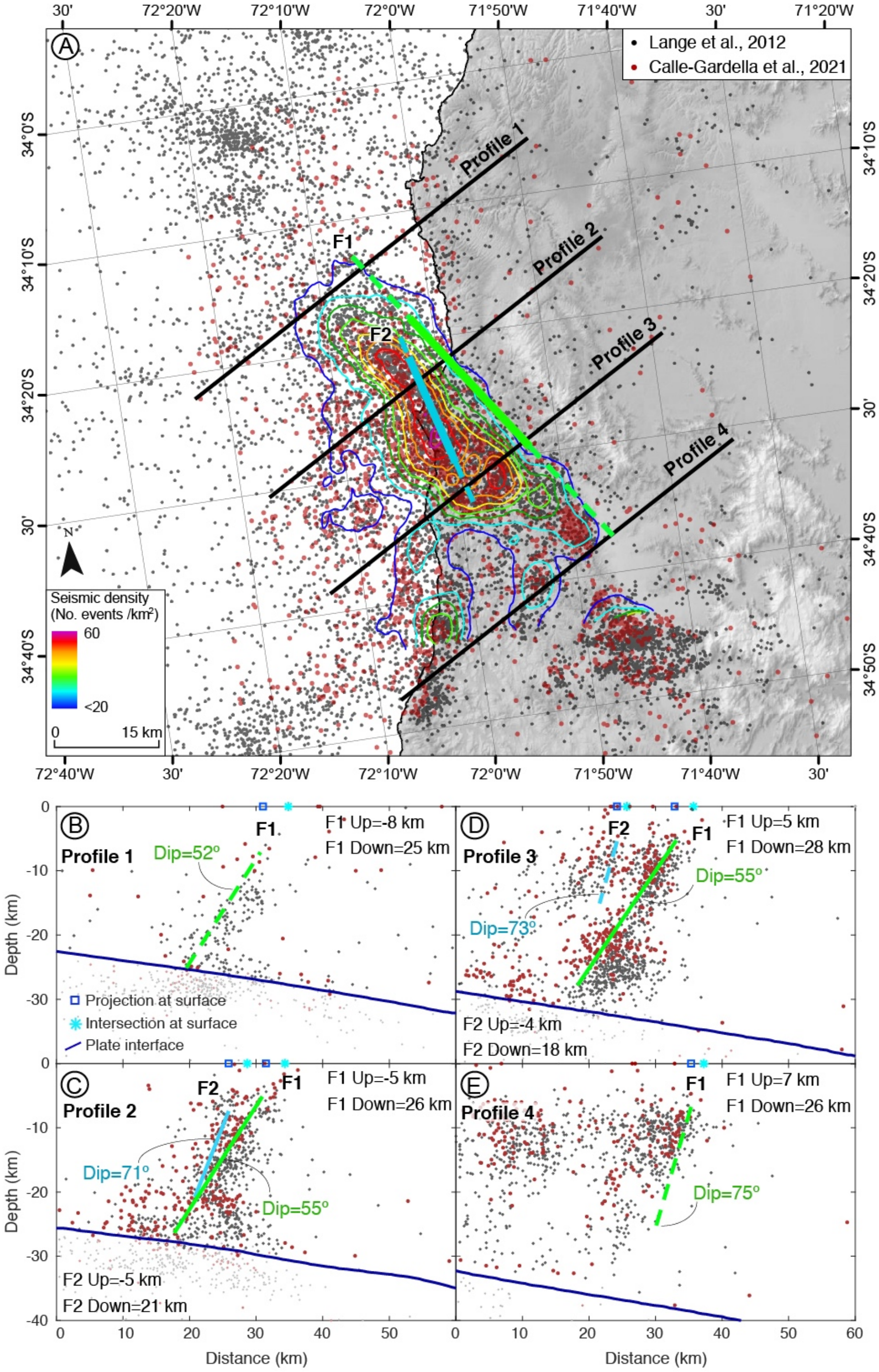

Figure S7: Fault geometry based on the distribution of aftershocks. A) Map with aftershock locations (Mw>1) [5,6] and profiles shown in B-E. Contours denote aftershock density. **B-E)** Profiles displaying the distribution of aftershocks and the location of faults

inferred from the alignment of aftershocks. Dark blue line shows the position of the plate interface[7]. Green and light-blue lines correspond to F1 and F2, respectively.

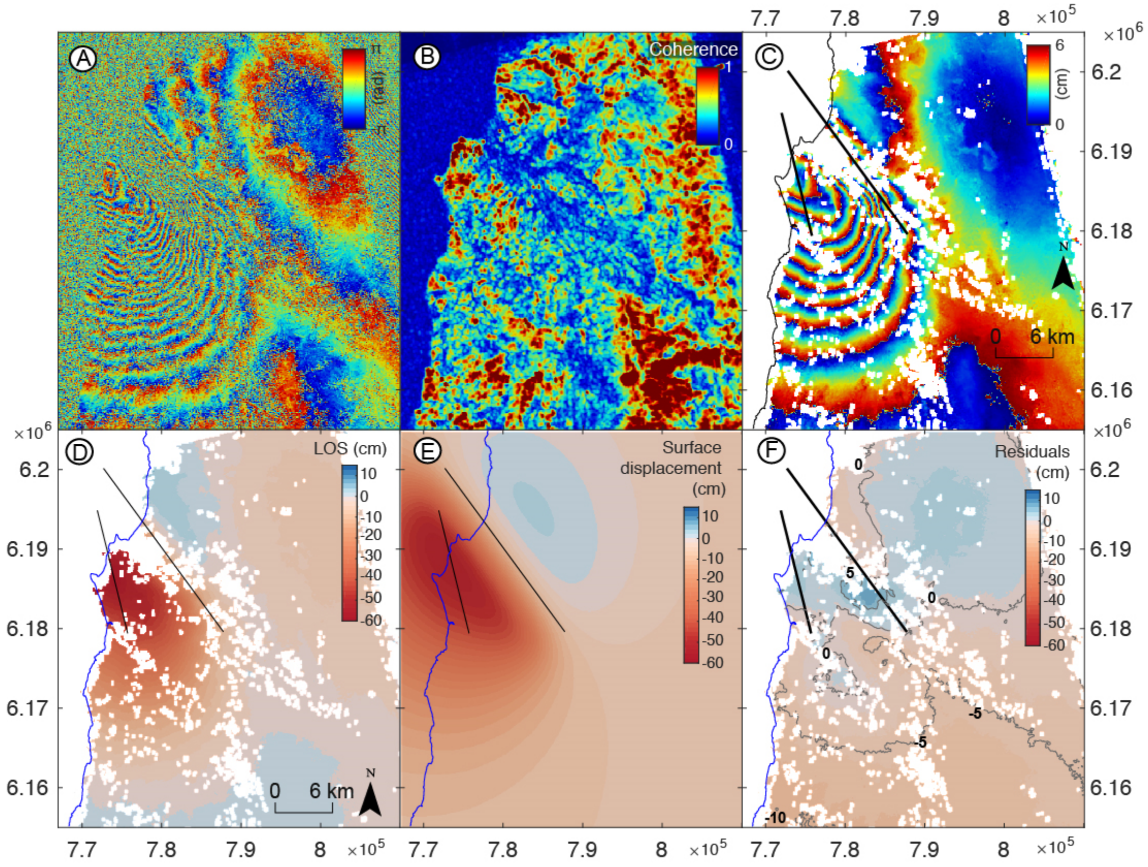

Figure S8: Processing and modelling of radar interferometric data. (A) Interferometric phase (4-look) before filtering and uncorrected for topographic distortion. **B)** Interferometric coherence (4-look); **C)** subsampled, unwrapped interferogram using a cyclic colour-scale, masked in areas where unwrapping was not achieved. **D)** Line Of Sight (LOS) displacements obtained from the unwrapped interferogram in C. **E)** Best-fit elastic forward model; **F)** residuals between LOS and best-fit elastic dislocation model.

Dislocation model	Parameter	Min value	Max value	Interval	No. of values
InSAR (85,918 models)	Up-dip F1 (km)	1.8	5.4	0.2	19
	Slip F1 (m)	0.6	1.5	0.05	19
	Up-dip F2 (km)	2.4	5.6	0.2	17
	Slip F2 (m)	0	0.65	0.05	14
Shoreline angles (116,964 models)	Up-dip F1 (km)	0	3.4	0.2	18
	Up-dip F2 (km)	0.2	3.6	0.2	18
	Slip rate F1 (m/ka)	0.32	0.68	0.02	19
	Slip rate F2 (m/ka)	0	0.36	0.02	19
Interpolated shoreline angles (116,964 models)	Up-dip F1 (km)	0	3.4	0.2	18
	Up-dip F2 (km)	0.2	3.6	0.2	18
	Slip rate F1 (m/ka)	0.32	0.68	0.02	19
	Slip rate F2 (m/ka)	0	0.36	0.02	19

Table S1: Parameters and ranges used for forward dislocation models.

Satellite	Heading	Direction	Start date	End date	Unit vector		
					E	N	Up
ENVISAT	-13.966	Ascending	2010/03/06	2010/04/10	0.332	-0.088	0.939

Table S2: Satellite orbit information and unit vector.

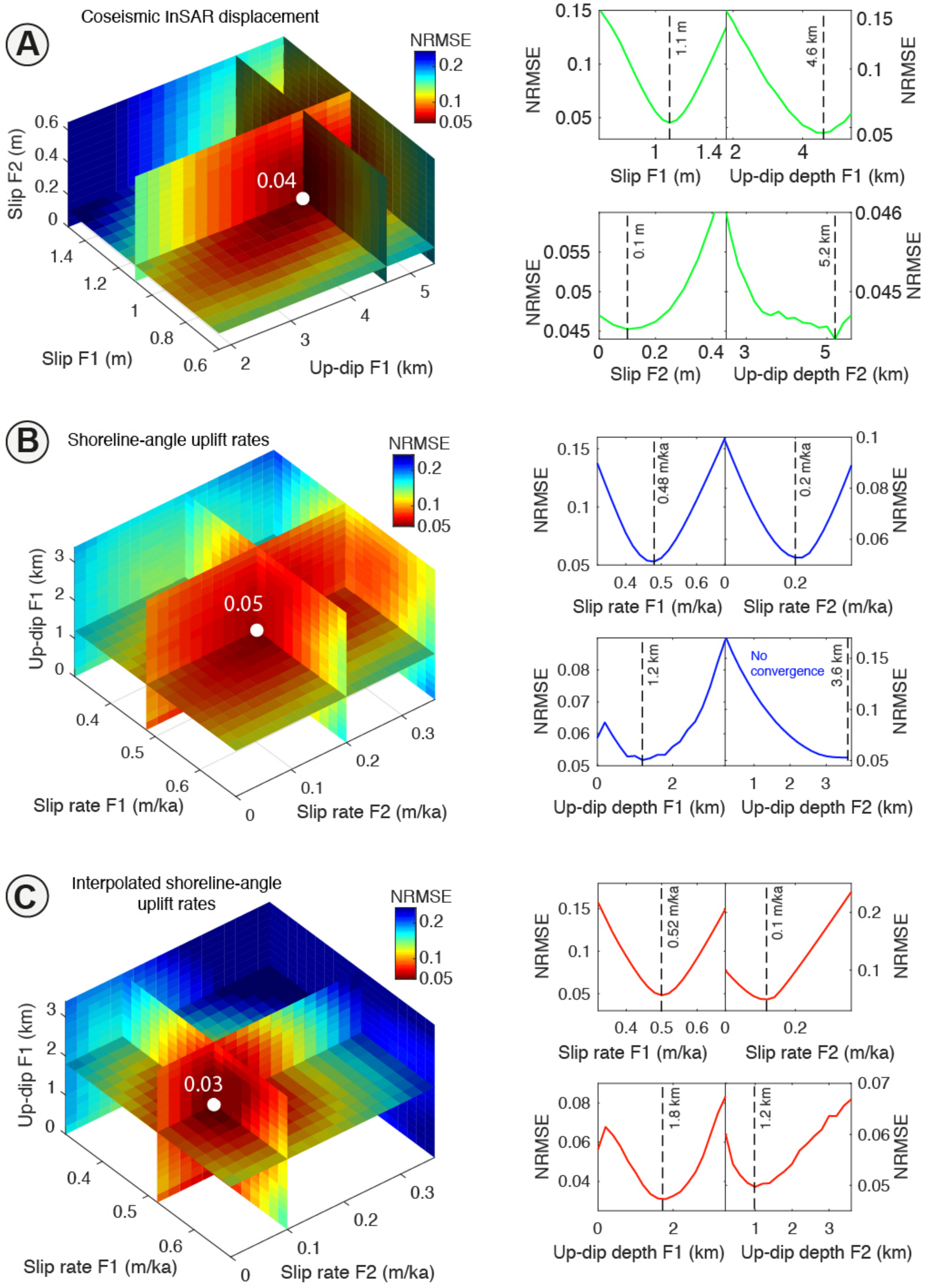

Figure S9: Best-fit values, 3D fence diagrams, and plots of NRMS distributions. A) Distribution of NRMSE values from 85,918 forward dislocation models reproducing

coseismic surface deformation estimated using InSAR data. Minimum NRMSE is denoted by a white dot. Fence diagrams display three of the four modelling parameters used. **B)** Distribution of NRMSE values from 116,964 dislocation models reproducing uplift rates estimated at measured shoreline-angle locations. **C)** Distribution of NRMSE values reproducing a smooth surface interpolated from shoreline-angle uplift rates. Note that plots in C exhibit a better convergence with lower NRMSE values compared to B. Black-dashed line in plots are the best-fit values.

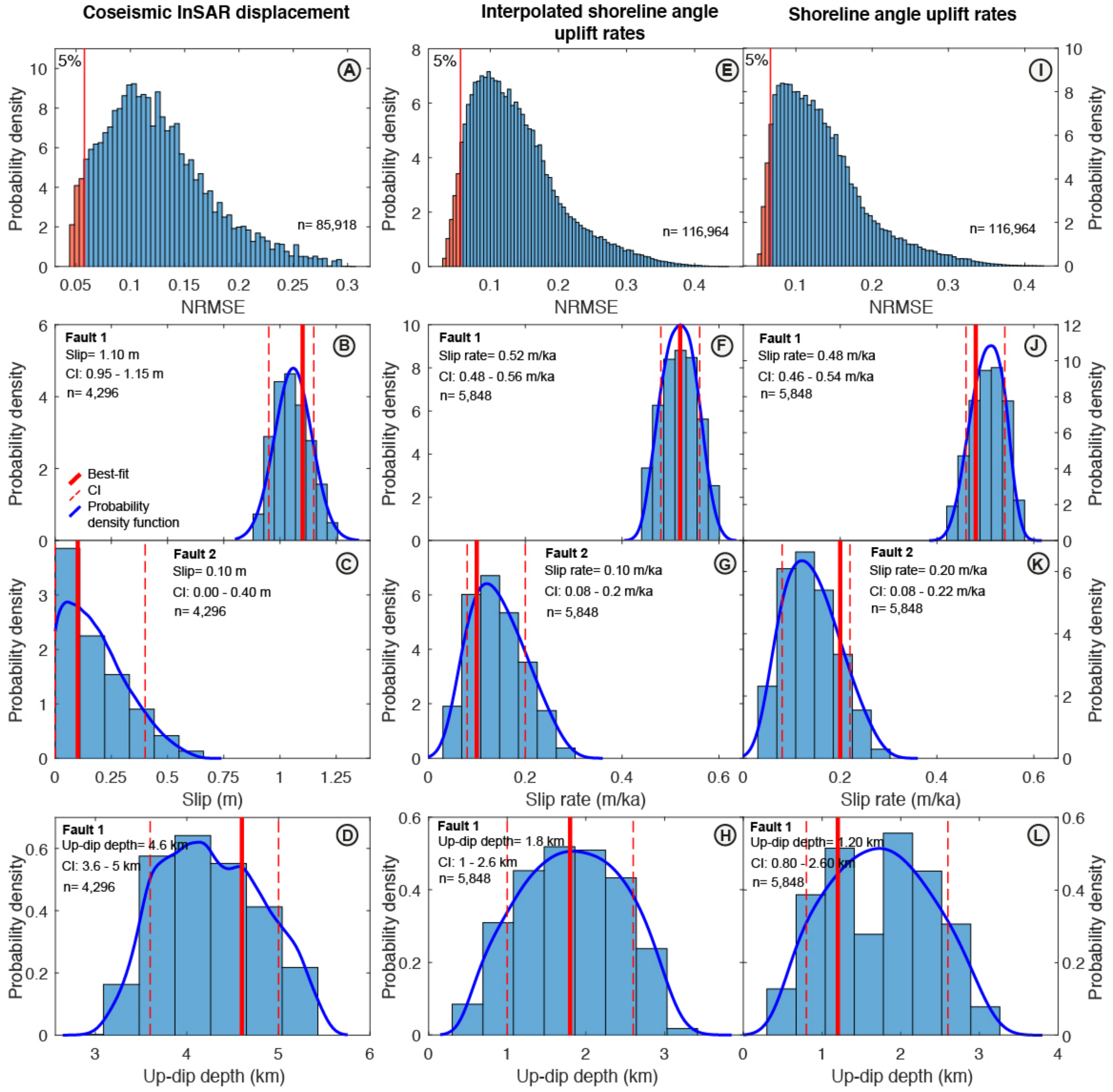

Figure S10: Distribution of NRMSE values and probability densities. A) Distribution of NRMSE values for coseismic deformation using InSAR LOS displacements. The orange area denotes the lower 5% tail used to calculate a probability-distribution function of slip-rate values; n is the number of model runs. **B** and **C)** Distribution of coseismic slip in Fault 1 (main branch of the PIF) and Fault 2 (secondary branch of the PIF) using the 5% NRMSE tail. The thick red line is the best-fit model obtained by minimizing NRMSE values, the red-dashed lines are the confidence intervals (CI) using the 90% of the distribution. **D)** Distribution of up-dip depths in F1 using the 5% NRMSE tail. **E)** Distribution of NRMSE values of dislocation models using interpolated uplift rates derived from shoreline angles

of marine terraces. **F, G** and **H)** distribution of fault-slip rates of F1 and F2 and up-dip depth of F1 from dislocation models based on uplift rates derived from interpolated shoreline angles of marine terraces using the 5% NRMSE tail. **I)** Distribution of NRMSE values of dislocation models using uplift rates derived from shoreline angles of marine terraces. **J**, **K** and **L)** are similar as F, G and H, but using uplift rates derived from shoreline angles of marine terraces.

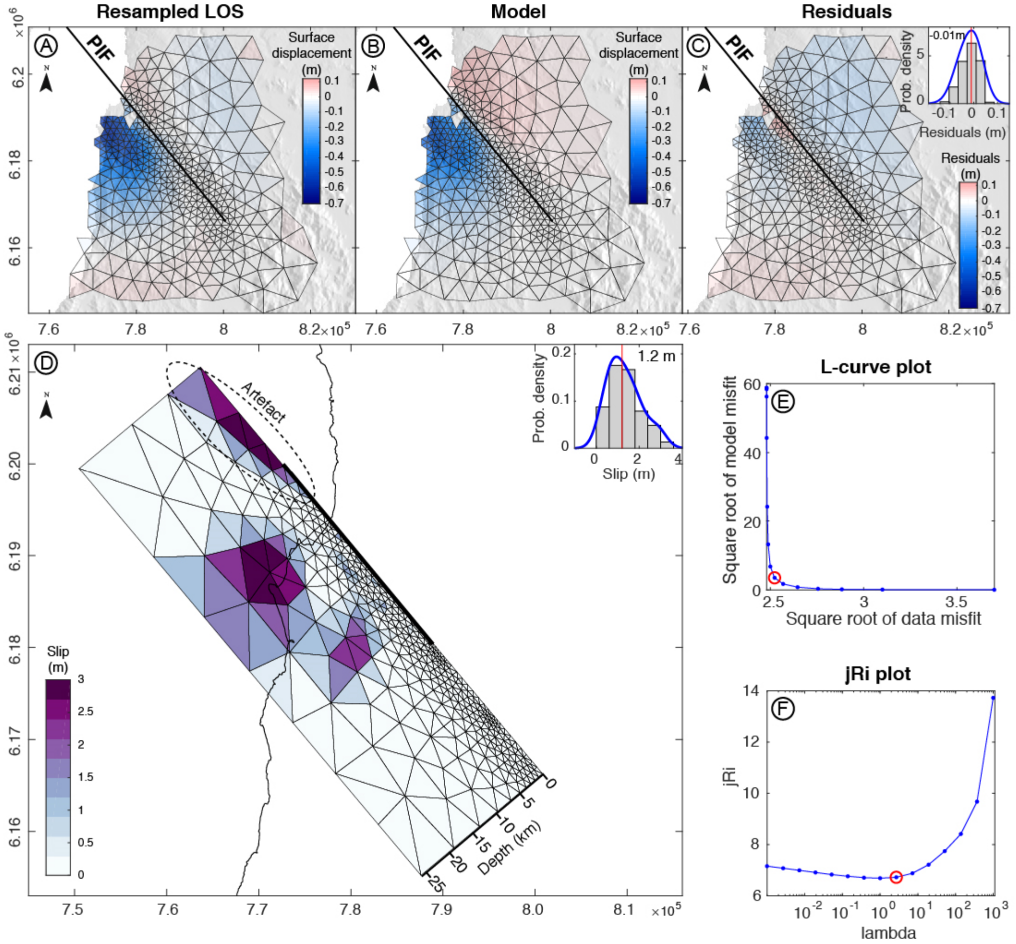

Figure S11: Slip inversion model. (A) Resampled LOS displacements **B)** Surface displacements of slip inversion model. **C)** Slip model residuals. Inset shows residual distribution with a median value of -0.01 m. **D)** Fault slip distribution map. Inset shows slip histogram within the area of fault F1. Black thick line shows the length of F1 used in forward models. Note that the median slip value of 1.2 m is similar to the 1.1 m slip estimated by the forward model. The slip distribution extends between ~6.5 km and ~26 km along an irregular slip patch at the central part of the fault. A small disconnected shallow slip patch located offshore is probably an artefact of the inversion due to the lack of data. **E)** The selected model regularization using the L-curve approach. The maximum curvature of L-curve (red circle) is consistent with the lambda value (2.68) obtained using the jRi method (red circle in F).

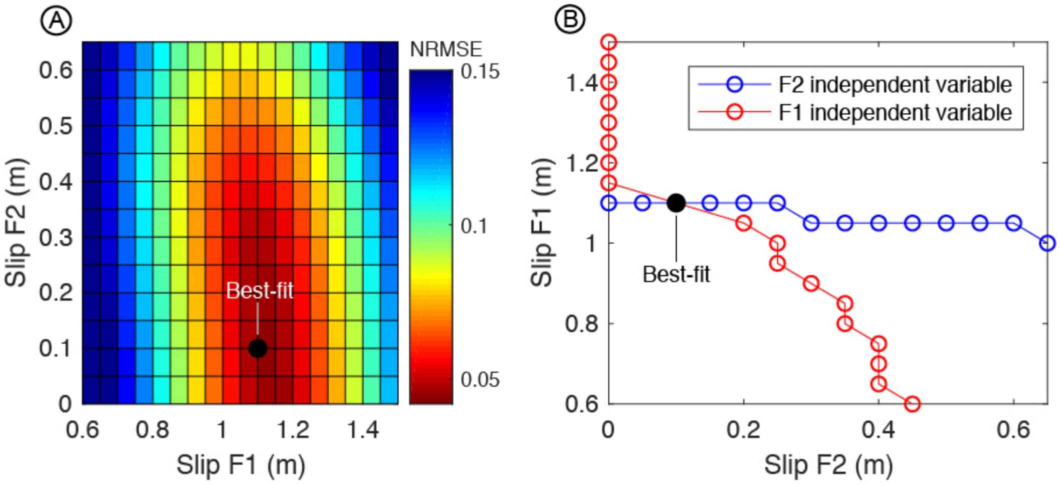

Figure S12: Evaluating trade-off between the slip of faults F1 and F2. A) Distribution of NRMSE for the slip ranges of F1 and F2. **B)** Dependency of slip of F1 and F2, circles represent the minimum NRMSE for each slip value. Notice that the slip of F1 varies ~10% when using the slip of F2 as independent variable. The black dot corresponds to best-fit estimated by minimization.

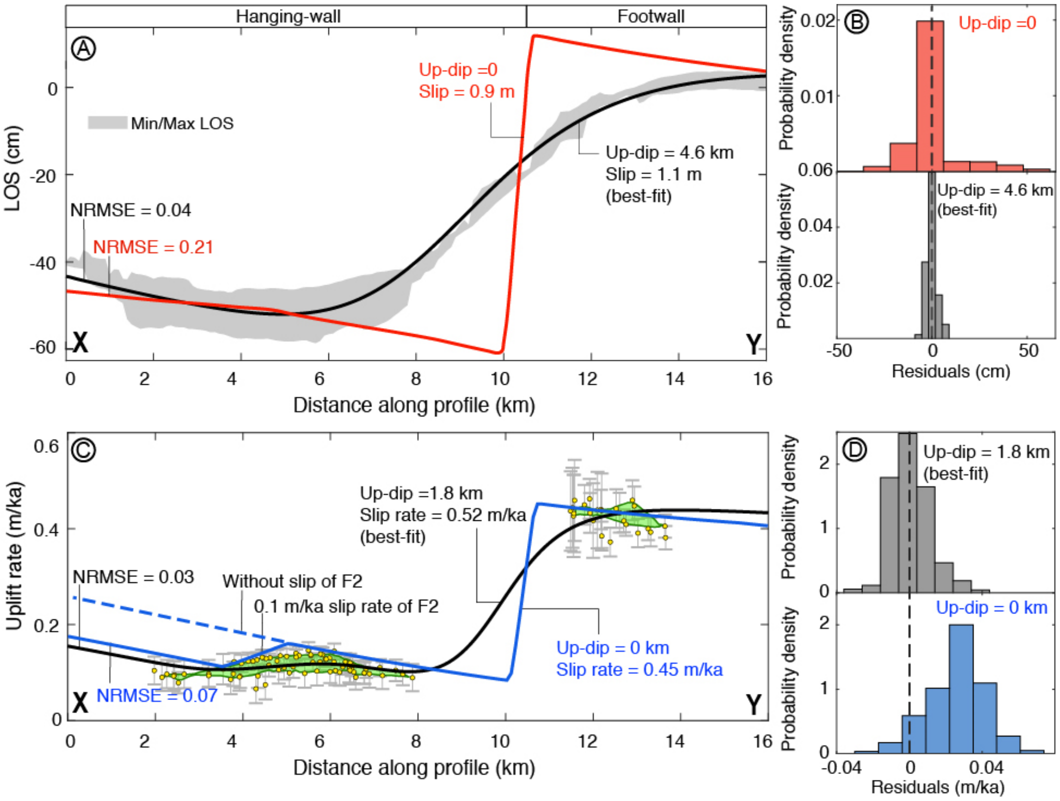

Figure S13: Modelling experiments using surface-breaching faults. A) Comparison between best-fit elastic model using the radar interferometry and an elastic model with arbitrary zero up-dip. **B)** Histograms of residuals; both histograms are centred at zero, but the residuals of the surface-breaching fault model display higher dispersion and positive bias (red histogram). **C)** Comparison between best-fit model using a smooth surface derived from interpolated shoreline-angle uplift rates (black line) and an elastic model using up-dip=0 (blue line). Notice that the former model reproduces the uplift rates in the footwall, but fails to adequately reproduce the uplift rates in the hanging wall (dashed blue line), even after adding slip along F2 (continuous blue line). **D**) Histograms of residuals of the best fit model (grey) and surface-breaching fault (blue). Notice that the centre of the blue histogram deviates from 0. NRMSE: normalized root mean-squared error.

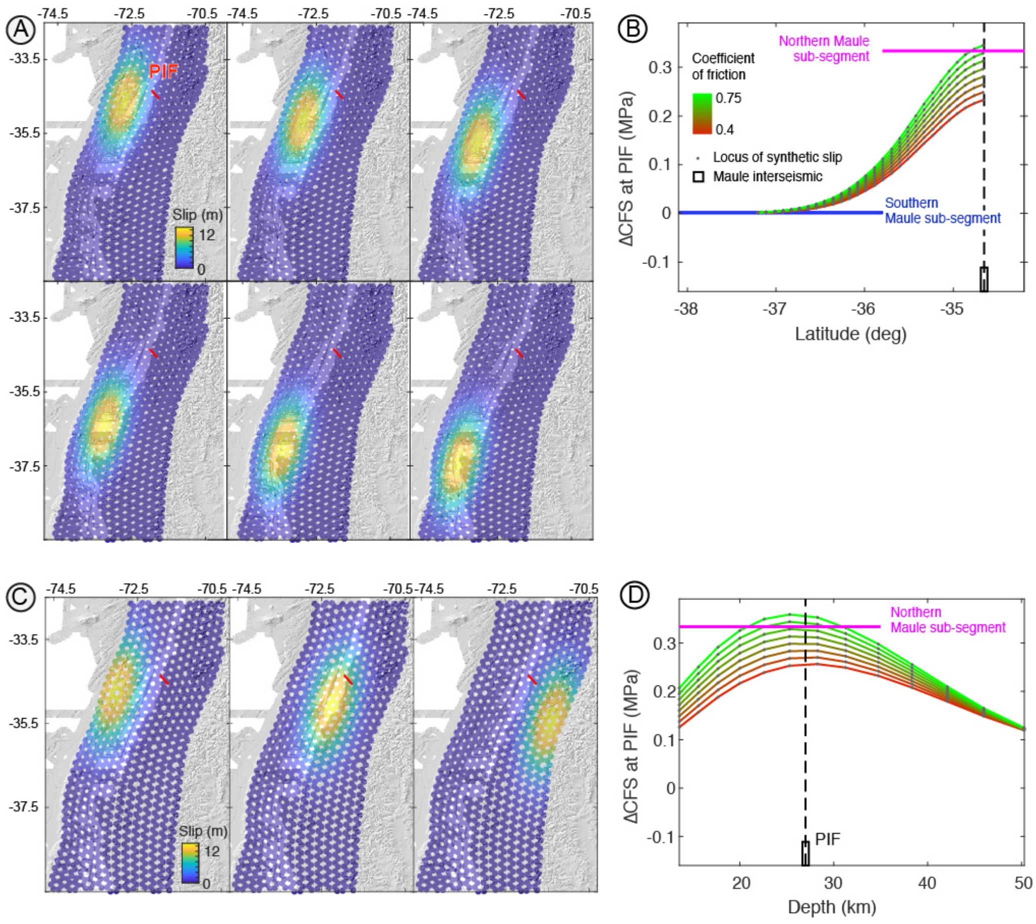

Fig. S14: Examples of synthetic coseismic-slip scenarios during megathrust earthquakes and ΔCFS on PIF. A) Along-strike synthetic slip scenarios within the Maule earthquake rupture zone. Red line denotes the PIF. **B)** Coulomb Failure Stress (ΔCFS) values on the PIF as normal receiver fault for the different slip scenarios in A and coefficients of friction. Only some of the synthetic slip scenarios are displayed in **A**. **C)** Across-strike slip scenarios for different down-dip depths at the northern part of the Maule rupture. **D)** ΔCFS values on the PIF as a normal receiver fault for the different slip scenarios in C and coefficients of friction. The black-dashed lines in B and D indicate the latitude and down-dip depth of the PIF, respectively; the black rectangle indicates the range of ΔCFS values during the pre-Maule earthquake interseismic period. The blue and pink lines in B indicate the extent of the southern and northern subsegments of the Maule earthquake. The pink line in D indicates the along-dip extent of the northern subsegment.

References

1 Hyppolito, T. *et al.* LP/HT metamorphism as a temporal marker of change of deformation style within the Late Palaeozoic accretionary wedge of central Chile. *Journal of Metamorphic Geology* **33**, 1003-1024 (2015).

2 Bookhagen, B. High resolution spatiotemporal distribution of rainfall seasonality and extreme events based on a 12-year TRMM time series. (UC Santa Barbara Geography, 2013).

3 Rohling, E. *et al.* Antarctic temperature and global sea level closely coupled over the past five glacial cycles. *Nature Geoscience* **2**, 500-504 (2009).

4 Bintanja, R., van de Wal, R. S. & Oerlemans, J. Modelled atmospheric temperatures and global sea levels over the past million years. *Nature* **437**, 125 (2005).

5 Lange, D. *et al.* Aftershock seismicity of the 27 February 2010 Mw 8.8 Maule earthquake rupture zone. *Earth and Planetary Science Letters* **317-318**, 413-425, doi:http://dx.doi.org/10.1016/j.epsl.2011.11.034 (2012).

6 Calle-Gardella, D., Comte, D., Farías, M., Roecker, S. & Rietbrock, A. Three-dimensional local earthquake tomography of pre-Cenozoic structures in the coastal margin of central Chile: Pichilemu fault system. *Journal of Seismology*, 1-13 (2021).

7 Hayes, G. P. *et al.* Slab2, a comprehensive subduction zone geometry model. *Science* **362**, 58-61 (2018).
