# Supplementary material for: The cryptic seismic potential of the Pichilemu blind fault in Chile revealed by off-fault geomorphology
Source: Nat Commun. 2022 Jun 11;13:3371. doi: 10.1038/s41467-022-30754-1 (PMC9188598; doi:10.1038/s41467-022-30754-1)
Supplement: Supplementary file 3 — Description of Additional Supplementary Files [file 41467_2022_30754_MOESM3_ESM.pdf]

Description of Additional Supplementary Files

File Name: Supplementary Data 1
Description: Google earth file (.kmz) that contains the results of the marine terrace mapping using the tool TerraceM. The attributes of each point in the file are:

SH:elev = Shoreline angle elevation (m)
SH:elev_err = Shoreline angle elevation error (m)
SH:age = Age of the terrace level (ka)
SH:age_err = Age error of the terrace level (ka)
SH:sl = Sea-level elevation when the marine terrace was formed (m)
SH:sl_err = Sea-level elevation error when the marine terrace was formed (m)
SH:up= Uplift rate (m/ka)
SH:up_err= Uplift rate error (m/ka)
